# Patients’ Satisfaction with E-Prescribing (Wasfaty) in Saudi Arabia: A Survey of Country-Level Implementation

**DOI:** 10.3390/healthcare10050806

**Published:** 2022-04-27

**Authors:** Dalia Almaghaslah, Abdulrhman Alsayari, Sokinh Almaghaslah, Haytham Alsanna

**Affiliations:** 1 Department of Clinical Pharmacy, College of Pharmacy, King Khalid University, Abha 61441, Saudi Arabia; 2 Department of Pharmacognosy, College of Pharmacy, King Khalid University, Abha 61441, Saudi Arabia; alsayari@kku.edu.sa; 3 Department of Family Medicine, Family Medicine Academy, Dammam 32210, Saudi Arabia; sukainaalmaghaslah@gmail.com; 4 Department of Emergency Medicine, Prince Sultan Hospital, First Health Cluster Estren Province, Mulija 32210, Saudi Arabia; halsanna@hotmail.com

**Keywords:** community pharmacy, 2030 Saudi vision, Wasfati, e-prescription, patient satisfaction

## Abstract

*Aim:* This study was conducted to assess patient satisfaction with the e-prescription service implemented by the Ministry of Health hospitals and primary healthcare centres in Saudi Arabia. *Methods:* The study used a cross-sectional approach. Data were collected using a random sampling technique, and an online questionnaire was distributed among the study population. A five-point Likert scale, ranging from 1 (not at all satisfied) to 5 (very satisfied), was used to assess patient satisfaction. *Results and Conclusions:* A total of 400 patients participated in the study. More than half (57.5%) of them were males, and approximately one-third were between the ages of 30 and 39. Aspects related to the pharmacy, i.e., accessibility of pharmacies in terms of numbers, location, and opening hours, as well as pharmacy facilities, including waiting area, counselling area, dispensing area and parking lots, were skewed towards 5 (very satisfied). Aspects related to pharmacy personnel, i.e., knowledge, skills, and competencies, as well as friendliness and approachability, were also skewed towards 5 (very satisfied). Factors related to patient experience with Wasfaty, the new service, as compared with old primary healthcare centres’ pharmaceutical services, such as the availability of pharmacists, procedures for refills, waiting time, privacy, and confidentiality, were also skewed towards 5 (very satisfied).

## 1. Introduction

The healthcare system in Saudi Arabia has changed drastically in the last few years [1]. The government–private partnerships that were given more attention in the Saudi 2030 Vision plan played a significant role in this change. Implementation of the Vision 2030 plan, in terms of focusing on primary preventative healthcare, as well as increasing the participation of the private sector in the delivery of these health services, was established by introducing an initiative that incorporated private community pharmacies in medication provision [2]. In 2018, an e-prescribing system called Wastfaty was launched in the government healthcare centres at both primary and secondary levels.

E-prescribing is defined as “the direct computer-to-computer transmission of electronic prescriptions (e-prescriptions) from the prescriber office to community pharmacies” [3]. The e-prescriptions are initiated by physicians and sent electronically to community pharmacies where patients can obtain their medications and other healthcare products free of charge. The initiative connects primary healthcare centres and hospitals to selected community pharmacies in various locations to allow easy access to the nearest pharmacy in the neighbourhood [4]. The services also aim to improve health spending efficiency and reduce medication waste, enhance medication availability, improve patient medication counselling and help prevent medication errors.

Community pharmacy is the largest private sector in the pharmacy field and employs the largest proportion of the pharmacy workforce in the country. In 2021, community pharmacies numbered 9026 around the country, employing 17,815 pharmacists [5]. Pharmacy ownership is limited to Saudi pharmacists registered with the Saudi Commission for Healthcare Specialities, the licensing body for healthcare professions. The maximum number of pharmacies that can be owned by a single pharmacist is 30 [1]. Community pharmacies are usually managed by one to two pharmacists or one pharmacist and an assistant. They usually operate between 8 and 12 h a day, 6 days a week [6]. Some pharmacies operate 24 h a day [7]. The Ministry of Health is the government body that regulates the community pharmacy sector [8].

Community pharmacies are required to fulfil the criteria that enable them to join the Wasfaty system. This includes a valid license for the premises, registration at Nupco (the leading provider of healthcare products to the government health sector in the country), the use of approved suppliers from the Wasfaty list, a valid pharmacist license from the Saudi Commission for Healthcare Specialties for all employees, a computer with Internet connection to access the online system, and a label printer. Currently, 3100 community pharmacies are registered and are serving 228 hospitals and 2069 primary healthcare centres in 174 cities around the country [9].

Similar information technology systems that facilitate the electronic transfer of prescriptions have been used in several countries around the world, including Sweden, Italy, the United States, and England [10]. For example, The National Health Services (NHS) in England incorporated community pharmacies in providing health services to the public by linking them with general practitioners (GPs) (primary healthcare centres). NHS contracts with local pharmacies for three levels of service: essential, advanced, and enhanced. The basic essential contract involves dispensing NHS prescriptions written by GPs; this is considered the main role of community pharmacies. The Electronic Prescription Service (EPS) allows electronic transfer of prescriptions from the GP surgeries to community pharmacies [11]. Community pharmacies are responsible for the medication supply, so patients with acute or chronic conditions can obtain their medications free of charge or incur a co-payment charge. NHS repays community pharmacies for the medication costs, as well as dispensing charges. Electronic submission of reimbursement is enabled through EPS to the NHS Business Service Authority [10].

Several benefits were identified with the use of EPS, including the fact that electronic prescriptions are accurate, complete, and readable, which reduces the chance of dispensing errors and omissions. It provides higher levels of security, regulates the repeat dispensing workload enabling patients to collect their medication on time, is a convenience to the patients since they do not have to wait for the paper prescription to be dispensed, and opens two-way communication between the prescriber and the pharmacist, allowing pharmacists to be more involved in patient-centred care. On the other hand, EPS is also associated with delays in receiving prescriptions and inaccurate prescriptions from physicians. An overwhelming number of prescriptions with unclear information may lead to delays in dispensing. A lack of formal training also affects the speed of dispensing [3]. 

A nationwide patient satisfaction survey assessing the newly implemented e-prescriptions system in Finland reported high satisfaction levels. The study concluded that no difficulties were encountered in purchasing medications, renewing prescriptions, or acting on behalf of someone in the pharmacy [12]. Similarly, a country-level evaluation of e-prescriptions in Sweden indicated that patients had a positive attitude towards e-prescribing, found e-prescribing to be safe and beneficial, and enabled a faster dispensing process [13].

National studies have concluded that replacing handwritten prescriptions with e-prescribing would have several benefits [14]. Hence, following the recent implementation of the Wasfaty e-prescribing system, a patient satisfaction assessment was needed to assess and further enhance the services provided. To the best to our knowledge, this is the first study to evaluate the Wasfaty e-prescribing system in Saudi Arabia. 

## 2. Methods

### 2.1. Study Design

The study used a cross-sectional anonymous self-administered online questionnaire. The study was conducted between January and March 2022. 

### 2.2. Sample Size and Sampling Procedure 

A non-probability convenience sampling method was used. Data were initially collected by two family medicine residents who interviewed patients at the end their visits and invited them to complete and submit the questionnaire electronically. Recruiting more participants was achieved by distributing the questionnaire through social media applications, including WhatsApp and Instagram community groups around Saudi Arabia. The sample size was determined based on the total population of Saudi Arabia (36,000,000) and determined by using a Raosoft sample size calculator (http://www.raosoft.com/samplesize.html (accessed on 15 March 2022)) with a predetermined margin of error of 5% and a confidence level of 95%. In order to minimise erroneous findings and increase study reliability, the target sample size was set at 370 patients. 

### 2.3. Data Collection Form

The structured questionnaire was adapted from previous studies with similar aims [15,16]. The questionnaire consisted of four domains: one section eliciting demographic and background information and three sections evaluating patients opinions towards the community pharmacy premises, pharmacy personnel, and e-prescription-related features using the five-point Likert scale. The data collection tool was initially prepared in the English language, then translated into the Arabic language. Translation validity was assured by retranslating the Arabic version of the survey into English (back translation). The study investigators, who were bilingual speakers of both languages, conducted the back translation. Face and content validity were carried out by a group of experts in the fields of clinical pharmacy. 

The online data collection tool was designed using Google forms. The reason for choosing Google forms was that the authors have successfully used it before in a country-level data collection among a Saudi population [17]. Hence, they were familiar with its features. A previous study confirmed that Google docs serve as an easy access, free of charge, and convenient platforms for questionnaire administration to clinical population. It also maintains the quality, security, and fidelity of the data [18]. 

The data collection tool was piloted with five participants, who were representative of the study population, to ensure the clarity of language and the questionnaire structure. The findings of the pilot study were not included in the final results. The questionnaire was reviewed and modified based on the feedback received in the pilot. The final questionnaire was distributed in Arabic. 

#### 2.3.1. Inclusion Criteria 

Patients who were eligible for free government healthcare services in Saudi Arabia, 18 years or older, having previously used Wasfaty services.

#### 2.3.2. Exclusion Criteria

Patients utilizing private healthcare services in Saudi Arabia, younger than 18 years, and patients who have never utilized the Wasfaty system. A filter question was used to exempt people who had no experience with the e-service. 

### 2.4. Statistical Analysis

The collected data were downloaded, entered, and analysed using the Statistical Package for Social Sciences (SPSS) version 26.0 for Mac. Demographic and background information were described in terms of frequencies. Community pharmacy premises features (two items), pharmacy personnel (two items) and e-prescription-related features (eight items) used a Likert scale ranging from 1 (Not at all satisfied) to 5 (very satisfied). The distribution of the scale was presented in percentages, as well as mean and SD. A mean value of ≥3 was considered skewed towards high satisfaction, while a mean value of <3 is considered skewed towards low satisfaction. The internal consistency and reliability of the scales was assessed using Cronbach’s alpha coefficient, with the minimum recommended level being 0.70 [1]. 

### 2.5. Ethics Consideration 

An ethical clearance was given by the Ethical Committee of Scientific Research, King Khalid University (ECM #2022-503).

## 3. Results

A total of 400 patients participated in the study. More than half (57.5%) of them were males, and around one-third were between the ages of 30 to 39. Slightly less than three-quarters (72%) were educated to university level and above. The vast majority were Saudi nationals (94.3%) who utilised Wasfaty services for an acute condition (Table 1).

The distribution of scale scores for all questions related to pharmacy personnel and services is presented in Table 2. All responses ranged from 1 (not at all satisfied) to 5 (very satisfied). Both items, i.e., accessibility to pharmacies in terms of numbers, location, and opening hours, as well as pharmacy facilities, including waiting area, counselling area, dispensing area and parking lots, were skewed towards 5 (very satisfied). Both items related to pharmacists, i.e., knowledge, skills, and competencies, as well as friendliness and approachability, were also skewed towards 5 (very satisfied). Eight items were related to patients’ experience with Wasfaty, comparing the new service with the old primary healthcare centres’ pharmaceutical services. All items, such as availability of pharmacists, procedures for refills, waiting time, privacy, and confidentiality were skewed towards 5 (very satisfied).

Table 3 presents the rate of satisfaction with aspects related to pharmacists, pharmacies, and Wasfaty service. These scales are treated as continuous variables ranging from 1 (not at all satisfied) to 5 (very satisfied). The mean value of the overall satisfaction scale with pharmacists was 3.5 and for pharmacies and Wasfaty was 3.3 services. All scales had Cronbach alpha coefficients greater than 0.7, indicating inter-item reliability.

## 4. Discussion 

This research was conducted to assess patient satisfaction with the e-prescribing system, Wasfaty, that connects Ministry of Health hospitals and primary healthcare centres to community pharmacies. The findings of the study indicated moderate satisfaction levels, with highest satisfaction given to pharmacy personnel in terms of their knowledge, communication skills, counselling skills, and therapy management, as well as openness and approachability (3.5/5). This national study on the Saudi population’s perceptions and attitudes towards community pharmacy services found high satisfaction levels with pharmacists’ commitment and communication skills.

Moderate satisfaction levels were also reported for the pharmacy premises-related features in terms of the facilities and accessibility, with a mean of (3.3/5). Similar findings were reported in a local study where patients had the same satisfaction levels regarding the counselling area and its privacy, but higher satisfaction levels were reported with waiting time (4.3/5) and waiting area (4.3/5) [19]. This could be because the study included only pharmacies that provide Wasfaty services which are free of charge to all nationals, so the demand on these pharmacies is likely to be higher, resulting in slower services.

Patient experience with Wasfaty scored the same mean, i.e., (3.3/5), as the pharmacy related aspects. Patients were satisfied with pharmacists’ availability with a mean of (3.6/5), with the given instructions (3.4/5) and with privacy and confidentially (3.5/5), but were less satisfied with medication availability (2.9/5) and communication between pharmacists and prescribers (3.07/5).

The transition to e-prescribing services has helped to engage the private sector in providing pharmaceutical services to the public through community pharmacies. Hence, it overcomes the issue of insufficient numbers of pharmacy personnel in primary healthcare centres [20]. As was previously reported, pharmacies in primary health care centres sometimes lack pharmacy personnel might be run by allied healthcare professional [21]. Hence, the study provided evidence that the pharmacists are not only more accessible now, but are also more approachable, friendly, and have the required knowledge, competencies, and skills. Therefore, the digitalisation of medication prescriptions has facilitated the achievement of the health strategic goals of the 2030 Vision, i.e., increasing access to healthcare, promoting primary preventative healthcare [2]. A national study indicated the general public were satisfied with community pharmacists and found them supportive, courteous, and helpful [19]. Another recent publication concluded that the enablers, which pharmacy students found relevant to choose community pharmacy career path were achieving the Vision 2030, being socially accountable, fulfilling their role in their local community, as well as educating the local society [1]. These findings are in line with this study’s results. Telepharmacy services where medications are delivered to patients via courier companies have successfully managed access to medication during COVID-19 pandemic [21,22].

Medication availability was the criterion that patients found least satisfactory. This issue was previously reported in a local study [19], and in a Polish study where elderly patients would rather have their medications dispensed from their local primary healthcare centres [23]. Overcoming this issue could be achieved by having medications delivered to the patients’ homes or the nearest community pharmacy, or by specifying which community pharmacy has the prescribed medication in stock. Similar findings regarding elderly patients’ preference for traditional paper-based medication prescriptions were reported in Finland, the USA, and Belgium [12,23,24]. This preference stems from a lack of access to or familiarity with the Internet and smart devices. The study in Poland reported the same problem, i.e., limited access to well-stocked pharmacies, especially those in rural areas. A strategy suggested to overcome this issue was having a family member, a neighbour, or a caregiver dispense the prescription in their behalf [25]. 

Communication between physicians and pharmacists was also seen to have room for improvement. This issue has been previously identified, particularly communication regarding suspected medication errors [26]. Communication might also be a problem because community pharmacies have different operation hours than primary healthcare centres, making it difficult to communicate with prescribers [27]. Establishing a medication therapy management program in community pharmacies might overcome these communication barriers between prescribers and pharmacists, where pharmacists have the authority to adjust treatment plans [27,28]. Policymakers who set the regulations for community pharmacies’ eligibility to join the Wasfaty program should probably encourage these pharmacies to further enhance the pharmacy facilities and pharmaceutical services, as well as provide training and continuing education programs for pharmacists. Big chain pharmacies that have branches around the country should be encouraged to join the Wasfaty program. Having more advanced pharmacies as part of the program might help in overcoming the identified core challenges, i.e., medication availability and communication issues. Additionally, pharmacists could assist in identifying and reporting the medications that are frequently unavailable; hence, the supply of these medication could be enhanced by the responsible authority (Nupco). 

This study has some limitations. The majority of participants were younger adults with higher education. Hence, the findings might not be generalizable to the entire population, especially the elderly, who are likely to have multiple chronic conditions and polypharmacy, yet comprise only 13% of the study population. Additionally, 70% of the sample visited community pharmacies for acute conditions, so they are not regular users of the service and probably reflect a one-time experience that might not be an accurate representation of the service pattern. The random distribution of the online questionnaire might have limited the variety of the sample, excluding older illiterate people from participation in the study. In addition, this study did not evaluate the Wasfaty e-prescribing system medication errors. Therefore, further study will be conducted to evaluate the effect of medication errors and extend the research. 

## 5. Conclusions 

The current study was conducted to assess the digitalization of medication prescriptions and the shifting of the receipt of medications from pharmacies located within primary healthcare centres to community pharmacies. The study suggested moderate satisfaction levels overall, with highest satisfaction given to pharmacy personnel in terms of their knowledge, communication skills, counselling skills, and therapy management, as well as openness and approachability. Pharmacy premises-related features in terms of the facilities’ availability and accessibility also scored high. Patient experience with Wasfaty was equally rated with their experience with the pharmacy-related aspects. Patients were satisfied with pharmacists’ availability, the given instructions, privacy, and confidentially, but were less satisfied with medication availability and communication between pharmacists and prescribers. 

## Figures and Tables

**Table 1:** Demographics.

	Frequency	Percentage (%)
**Gender**		
Male	230	57.5
Female	170	42.5
**Age**		
18–29	125	31.3
30–39	138	34.5
40–49	85	21.3
>50	52	13
**Education**		
High school and below	112	28
University education and above	288	72
**Nationality**		
Saudi	377	94.3
Non-Saudi	23	5.8
**Reason for visit**		
Acute condition	283	70.8
Chronic condition	117	29.3

**Table 2:** Distribution of factors affecting patients experience with e-prescription service (Wasfaty) ranging from 1 (not at all satisfied) to 5 (very satisfied).

Criterion	Not at All Satisfied	Not Very Satisfied	Moderately Satisfied	Satisfied	Very Satisfied	Skew	Mean	SD
	Frequency, Distribution of Responses (%)							
**Aspects related to the pharmacy**								
**Accessibility:** Location, number of pharmacies in your area, and working hours	52, 13	40, 10	86, 21.5	128, 32	94, 23.5	−0.54	3.4	1.3
**Pharmacy facilities:** Waiting area, Counselling space, dispensing area, and parking lots	70, 17.5	42, 10.5	112, 28	113, 28.2	63, 15.8	−0.31	3.2	1.1
**Aspects related to pharmacy personnel**								
Knowledge and skills such as counselling skills, communication skills, and medication therapy management	41, 10.3	42, 10.5	102, 25.5	137, 34.3	78, 19.5	−0.54	3.4	1.2
Approachability and friendliness of pharmacists and support staff	40, 10	26, 6.5	89, 22.3	147, 36.8	98, 24.5	−0.76	3.6	1.2
**Experience with this new service, and compare it with the old PHC pharmaceutical service**								
Availability of prescribed medications	94, 23.5	47, 11.8	105, 26.3	97, 24.3	57, 14.2	−0.11	2.9	1.3
Availability of pharmacists	35, 8.8	24, 6	105, 26.3	155, 38.8	81, 20.3	−0.74	3.6	1.14
Procedures for Prescription Refills	58, 14.5	53, 13.3	108, 27	116, 29	65, 16.3	−0.31	3.2	1.27
Medication delivery service (during COVID)	52, 13	40, 10	122, 30.5	111, 27.8	75, 18.8	−0.39	3.3	1.25
Privacy and confidentiality	41, 10.3	38, 9.5	86, 21.5	134, 33.5	101,25.3	−0.64	3.5	1.24
Waiting time	54, 13.5	39, 9.8	105, 26.3	125, 31.3	77, 19.3	−0.46	3.3	1.24
Clarity of instructions on medications (verbal/written)	52, 13	35, 8.8	85, 21.3	148, 37	80, 20	−0.62	3.4	1.26
Communication between physicians and pharmacists	85, 21.3	43, 10.8	100, 25	102, 25.5	70, 17.5	−0.21	3.07	1.3

**Table 3:** Distribution and internal consistency of factors for affecting patients experience with e-prescription service (Wasfaty) scale.

Description of Scale	≤1	≤2	≤3	≤4	≤5	Skew	Mean	SD	Cronbach α
Pharmacists	7.5	15	37.3	75.5	100	−0.66	3.5	1.14	0.856
Pharmacy	10.5	22.5	43.5	80	10.5	−0.47	3.3	1.21	0.879
Service	5	14.2	40.8	78.3	100	−0.35	3.3	1.08	0.944

## Data Availability

The data presented in this study are available on request from the corresponding author.

## References

[1] Almaghaslah D., Alsayari A., Almanasef M., Asiri A. (2021). A Cross-Sectional Study on Pharmacy Students’ Career Choices in the Light of Saudi Vision 2030: Will Community Pharmacy Continue to Be the Most Promising, but Least Preferred, Sector?. Int. J. Environ. Res. Public Health.

[2] Almaghaslah D., Alsayari A. (2021). Using a Global Systematic Framework Tool to Identify Pharmacy Workforce Development Needs: A National Case Study on Saudi Arabia. Risk Manag. Healthc. Policy.

[3] Odukoya O.K., Chui M.A. (2012). Relationship between E-Prescriptions and Community Pharmacy Workflow. J. Am. Pharm. Assoc..

[4] Juffali L.A.A., Knapp P., Al-Aqeel S., Watson M.C. (2019). Medication safety problems priorities in community pharmacy in Saudi Arabia: A multi-stakeholder Delphi study using the human factors framework. BMJ Open.

[5] Ministry of Health (2021). Statistical Yearbook-Chapter II—Health Resourses.

[6] Almanasef M., Almaghaslah D., Kandasamy G., Vasudevan R., Batool S. (2021). Involvement of community pharmacists in public health services in Asir Region, Saudi Arabia: A cross-sectional study. Int. J. Clin. Pract..

[7] Alsayari A., Almghaslah D., Khaled A., Annadurai S., Alkhairy M.A., Alqahtani H.A., Alsayed B.A., Alasiri R.M., Assiri A.M. (2018). Community Pharmacists’ Knowledge, Attitudes, and Practice of Herbal Medicines in Asir Region, Kingdom of Saudi Arabia. Evid.-Based Complement. Altern. Med..

[8] Almaghaslah D., Alsayari A., Abumelha S. (2021). Integrating simulation into Introductory Pharmacy Practice Experience (IPPE): A case study from Saudi Arabia. Trop. J. Pharm. Res..

[9] (2022). Wasfaty. https://wasfaty.sa/.

[10] Goundrey-Smith S. (2018). The Connected Community Pharmacy: Benefits for Healthcare and Implications for Health Policy. Front. Pharm..

[11] Saramunee K., Chaiyasong S., Krska J. (2011). Public health roles for community pharmacy: Contrasts and similarities between England and Thailand. Isan J. Pharm. Sci. IJPS.

[12] Lämsä E., Timonen J., Ahonen R. (2018). Pharmacy Customers’ Experiences with Electronic Prescriptions: Cross-Sectional Survey on Nationwide Implementation in Finland. J. Med. Internet Res..

[13] Hammar T., Nyström S., Petersson G., Astrand B., Rydberg T. (2011). Patients satisfied with e-prescribing in Sweden: A survey of a nationwide implementation. J. Pharm. Health Serv. Res..

[14] Alqahtani S.S. (2021). Community Pharmacists’ Opinions towards Poor Prescription Writing in Jazan, Saudi Arabia. Healthcare.

[15] Aziz M.M., Ji W., Masood I., Farooq M., Malik M.Z., Chang J., Jiang M., Atif N., Fang Y. (2018). Patient Satisfaction with Community Pharmacies Services: A Cross-Sectional Survey from Punjab; Pakistan. Int. J. Environ. Res. Public Health.

[16] Saramunee K., Krska J., Mackridge A., Richards J., Suttajit S., Phillips-Howard P. (2014). How to enhance public health service utilization in community pharmacy? General public and health providers’ perspectives. Res. Soc. Adm. Pharm..

[17] Almaghaslah D., Alsayari A., Kandasamy G., Vasudevan R. (2021). COVID-19 Vaccine Hesitancy among Young Adults in Saudi Arabia: A Cross-Sectional Web-Based Study. Vaccines.

[18] Rayhan R.U., Zheng Y., Uddin E., Timbol C., Adewuyi O., Baraniuk J.N. (2013). Administer and collect medical questionnaires with Google documents: A simple, safe, and free system. Appl. Med. Inf..

[19] Alotaibi N.H., Alzarea A.I., Alotaibi A.M., Khan Y.H., Mallhi T.H., Alharbi K.S., Alruwaili N.K., Alanazi A.S., Hassan A., Alotaib B.S. (2021). Exploring satisfaction level among outpatients regarding pharmacy facilities and services in the Kingdom of Saudi Arabia; a large regional analysis. PLoS ONE.

[20] Almaghaslah D., Alsayari A., Asiri R., Albugami N. (2019). Pharmacy workforce in Saudi Arabia: Challenges and opportunities: A cross-sectional study. Int. J. Health Plan. Manag..

[21] Khan A., Alahmari A., Almuzaini Y., Alturki N., Aburas A., Alamri F.A., Albagami M., Alzaid M., Alharbi T., Alomar R. (2021). The role of digital technology in responding to COVID-19 pandemic: Saudi Arabia’s experience. Risk Manag. Healthc. Policy.

[22] Hassounah M., Raheel H., Alhefzi M. (2020). Digital response during the COVID-19 pandemic in Saudi Arabia. J. Med. Internet Res..

[23] Hazazi A., Wilson A. (2021). Leveraging Electronic Health Records to Improve Management of Noncommunicable Diseases at Primary Healthcare Centres in Saudi Arabia: A Qualitative Study. BMC Fam. Pract..

[24] Lapane K.L., Dubé C., Schneider K.L., Quilliam B.J. (2007). Patient Perceptions Regarding Electronic Prescriptions: Is the Geriatric Patient Ready?. J. Am. Geriatr. Soc..

[25] Suykerbuyk L., Robbrecht M., De Belder S., Bastiaens H., Martinet W., De Loof H. (2018). Patient Perceptions of Electronic Prescriptions in Belgium: An Exploratory Policy Analysis. Pharmacy.

[26] Wrzosek N., Zimmermann A., Balwicki Ł. (2021). A Survey of Patients’ Opinions and Preferences on the Use of E-Prescriptions in Poland. Int. J. Environ. Res. Public Health.

[27] Wormser G.P., Erb M., Horowitz H.W. (2016). Are Mandatory Electronic Prescriptions in the Best Interest of Patients?. Am. J. Med..

[28] Albabtain B., Hadi M.A., Bawazeer G., Alqahtani A., Bahatheq A., Alhossan A., Cheema E. (2021). Evaluation of a community pharmacy-based medication therapy management programme: A study protocol of a pilot randomized controlled trial with an embedded qualitative study. Saudi Pharm. J..

